# Rapid and direct detection of hepatitis E virus in raw pork livers by recombinase polymerase amplification assays

**DOI:** 10.3389/fcimb.2022.958990

**Published:** 2022-09-05

**Authors:** Kairui Wang, Jinfeng Wang, Cang Zhou, Xiaoxia Sun, Libing Liu, Xiangdong Xu, Jianchang Wang

**Affiliations:** ^1^ School of Public Health, Hebei Medical University, Shijiazhuang, China; ^2^ Key Laboratory of Environment and Human Health, Hebei Medical University, Shijiazhuang, China; ^3^ Food Microbiology and Animal Quarantine Laboratory, Technology Center of Shijiazhuang Customs, Shijiazhuang, China

**Keywords:** hepatitis E virus, ORF2, qRT-RPA, LFB RT-RPA, isothermal

## Abstract

Hepatitis E virus (HEV) is a zoonotic pathogen that causes global hepatitis E. Outbreaks of hepatitis E are directly linked to the consumption of pork liver products. Herein reverse transcription recombinase polymerase amplification assays targeting the *ORF2* gene were developed for the rapid detection of HEV by integrating the fluorescence detection platform (qRT-RPA) and the visible lateral flow biosensor by naked eyes (LFB RT-RPA). The qRT-RPA assay effectively detected HEV RNA with a limit of detection (LOD) of 154 copies/μl (95%CI: 126–333 copies/µl) in Genie III at 41°C for 20 min. Besides this, the LFB RT-RPA detected the HEV RNA with a LOD of 24 copies/μl (95%CI: 20–57 copies/µl) in an incubator block at 41°C for 20 min. The developed RT-RPA assays also showed good specificity for HEV, with no cross-reactions with any of the other important swine pathogens examined in this work. The performance of the developed RT-RPA assays was validated on 14 HEV RNA-positive and 66 HEV RNA-negative raw pork liver samples identified by a previously described qRT-PCR. Consequently, 11 and 12 samples were HEV RNA-positive as detected by the qRT-RPA and the LFB RT-RPA, respectively. Compared to qRT-PCR, the qRT-RPA and LFB RT- RPA assays revealed a coincidence rate of 96.3 and 97.5% as well as a Kappa value of 0.858 and 0.908, respectively. These results ascertain that the developed RT-RPA assays are effective diagnostic tools for the point-of-care detection of HEV in resource-limited settings.

## Introduction

Hepatitis E virus (HEV) is a small, non-enveloped, and single-stranded positive RNA virus that is 7.2 kb in size and belongs to the *Hepeviridae* family (Wang et al., 2021). HEV is an emerging zoonotic virus transmitted through the fecal–oral route and causes acute or chronic infections (von Wulffen et al., 2018). Its symptoms include mild fever, nausea and vomiting, fatigue and anorexia, abdominal pain, and dark urine. HEV also contributes to extrahepatic manifestations, such as neurological, hematological, and renal conditions (Webb and Dalton, 2019). HEV infection causes self-limiting hepatitis with a mortality rate of approximately 0.2–4%, except for pregnant women and patients with chronic liver disease with a mortality rate of up to 30% (Hennechart-Collette et al., 2021; Raji et al., 2021).

HEV can adapt to a variety of hosts, including pigs, wild boars, deer, rabbits, and camels (Treagus et al., 2021). These animals act as potential reservoirs of HEV, causing the indirect transmission of HEV as well as promoting genetic variation and HEV evolution. Pigs are the primary reservoirs of HEV (Ukuli and Mugimba, 2017). Infected pigs may have no apparent clinical symptoms; however, viruses are shed from their feces or urine route at the early stages of infection. If these excreta are not properly handled, pigs in the same pen infect each other through repeated and direct contact with excreta, thus aggravating HEV infection (Salines et al., 2017). The virus persists in all age groups, from weaners to fatteners (Berto et al., 2012; Jackova et al., 2021). Therefore, HEV transmission is primarily attributed to the consumption of contaminated raw or undercooked pork products, therefore presenting a potential risk to public health. Additionally, excreta harboring HEV can pollute nearby irrigation and coastal waters, resulting in contaminated crops or seafood and thereby increasing the risk of human infection (Salines et al., 2017).

Real-time reverse transcriptase PCR (qRT-PCR) is the most common method for HEV detection (Mykytczuk et al., 2017). This technology involves denaturation, annealing, and the subsequent extension of the target gene. It requires the use of an accurate and stable thermocycler as well as a reliable power suppl and is only limited to well-equipped laboratories (Lobato and O'Sullivan, 2018). Recombinase polymerase amplification (RPA) is a novel enzymatic-based DNA amplification technology where primer annealing and elongation are mediated by different enzymes at constant temperature (Daher et al., 2016). The amplification proceeds at constant temperatures between 37 and 42°C and then typically run to completion within 30 min (Geng et al., 2019). The reaction starts when a recombinase protein uvsX binds to primers, forming a recombinase–primer complex. Subsequently, the complex recombines with homologous sequences in double-stranded DNA, hence forming a D-loop structure and initiating a strand exchange reaction. To prevent primer dissociation, the displaced DNA strand is stabilized by a single-stranded binding protein. Eventually, the amplification is initiated by a strand-displacing DNA polymerase (Piepenburg et al., 2006; Hill-Cawthorne et al., 2014). The RPA amplification products can be detected through gel electrophoresis, probe-based fluorescence monitoring, and lateral flow dipsticks (Liu et al., 2019). Therefore, recombinase polymerase amplification has a significant potential in an underequipped laboratory or in point-of-care diagnostics (Li et al., 2018; Davi et al., 2019).

For the rapid detection of HEV, we developed reverse transcription RPA assays targeting the *ORF2* gene of HEV by combining fluorescence detection platform (qRT-RPA) and visible lateral flow biosensor by naked eyes (LFB RT-RPA). Thereafter, the assays were validated using raw pork livers.

## Materials and methods

### Viruses and samples

The pET28a-ORF2 construct containing the HEV *ORF2* gene (2,025 bp) was artificially synthesized by Cencefe (Cencefe Biotech, Jiangsu, China) based on the reference sequences of HEV (accession number: AY594199). Pseudorabies virus (PRV, strain SH151218), porcine circovirus-2 (PCV-2, strain HB-MC1), respiratory and reproductive syndrome virus (PRRSV, strain HB-Xl), classical swine fever virus (CSFV, strain AV1412), porcine parvovirus (PPV, strain BJ-2), and the denatured cell-free extracts of foot-and-mouth disease (FMDV, serotype O) were kept in the laboratory.

A total of 626 raw pork liver samples were collected from different regions in Hebei Province between April 2021 and March 2022, *i*.*e*., 213 from the different retail markets, 300 from three different pig slaughterhouses, and 113 from the different Bio-Safety Disposal Centers for Dead Livestock and Poultry (BsDC) in Hebei Province. Total RNA extracted from 626 raw pork livers was detected using the previously described qRT-PCR assay (Qiao et al., 2008). Among these samples, 14 were HEV-positive with Ct values ranging from 30.23 to 37.46 and were confirmed as genotype 4d by sequencing the PCR product of a nested PCR assay described (Huang et al., 2002). The clinical performance of the RPA assays was established by testing 14 positive and 66 randomly selected negative samples.

### Viral and sample DNA/RNA extraction

PRV, PCV2, PRRSV, CSFV, PPV, and FMDV viral DNA or RNA were extracted using the TIANamp Virus genomic RNA kit (Tiangen Biotech, Beijing, China) following the manufacturer’s instructions. For the liver samples, approximately 100 mg of each sample was transferred into a 1.5-ml sterilized centrifuge tube with five 3.0-mm grinding beads and 1 ml of phosphate-buffered saline and then homogenized at 30 frequency/s for 5 min with an MM400 grinding mill (Retsch, Haan, Germany). After 12,000×*g* centrifugation at 4°C for 10 min, 200 µl supernatant was collected for viral RNA extraction using the Magnetic Viral DNA/RNA Extraction Kit (TIANLONG, Xian, China) with an np986-C Nucleic Acid Extraction System (TIANLONG, Xian, China) following the manufacturer’s instructions. The nucleic acids were stored at −80°C for subsequent analysis.

### Generation of standard RNA

The pET28a-ORF2 was first linearized with *BamHI* (TaKaRa, Dalian, China) and then transcribed into RNA using T7RiboMAX™Express Large Scale RNA Production System (Promega, USA). Subsequently, the residue plasmid DNA was eliminated using RNase-Free DNaseI (Tiangen, Beijing, China), and the transcribed RNA was purified using the RNA Clean Kit (Tiangen, Beijing, China). The *in vitro* transcribed standard RNA was quantified as 3.4 × 10^8^ copies/μl using a previously established RT-ddPCR assay in our laboratroy. Afterward, 10-fold serial dilutions of standard HEV RNA ranging from 3.4 × 10^6^ to 3.4 × 10^0^ copies/μl were prepared for further studies.

### Design of primers and probes

To design the primers for the RT-RPA assays, 21 genomic sequences for different HEV strains available in GenBank (GenBank accession numbers: HEV-1: AF185822, D11093, and X98292; HEV-2: M74506 and KX578717; HEV-3: AB089824, AB091394, and AB189070; HEV-4a: AB197673 and EF077630; HEV-4b: DQ279091 and EU676172; HEV-4c: AB099347 and AB16717; HEV-4d: AY594199, GU361892, and KC163335; HEV-4e: AB074915; HEV-4g: AB108537; and HEV-4h: GU188851) were examined to identify the conserved regions in the *ORF2* gene using the DNASTAR software (DNASTAR Inc., Madison, WI, USA).

The primers were screened in triplicates based on the TwistAmp™ amplification guidelines (TwistDx Ltd., Cambridge, UK) to obtain primers with the best performance. In the primary candidate screening, three forward and four reverse candidate primers were designed in the conserved region of the HEV *ORF2* gene (**Table 1**). The different combinations of candidate primers were screened using the ZC BioScience™ basic kit (ZC BioScience, Hangzhou, China) and then analyzed using 2% gel electrophoresis. Primer pairs with the most product yield and less product/noise ratio were selected for secondary candidate screening, and the probes HEV-RPA-P1 and HEV-RPA-P3 were designed. Thereafter, the primers were screened by keeping the primer length unchanged while moving in one base increment around the selected primers. Therfore, five forward (HEV-RPA-F301-F305) and four reverse primers (HEV-RPA-R301-R304) were obtained. The newly obtained primers were screened using the ZC BioScience™ exo kit (ZC BioScience, Hangzhou, China). Considering that the position of the forward primer could only be moved within a small range (more mutations and continuous repeating bases around this region), all available primers in this region were considered, and the tertiary candidate screening was not performed. Eventually, the reverse primer was refined by adding and subtracting bases from the 3′ end of the primer HEV-RPA-R301, and four reverse primers (HEV-RPA-R3001-R3004) were obtained. The newly obtained primers were screened similarly to the previous round. All primers and probes in **Table 1** were synthesized by Geneary (GenerayBiotech, Shanghai, China).

**Table 1:** Sequences of the primers and probes for the HEV qRT-RPA, LFB RT-RPA, and qRT-PCR assays.

Primers and probes	Sequence 5′-3′	Primer/probe location	Source
HEV-RPA-F1	ACCCTGTTTAATCTTGCTGACACGCTKCTCGG	6306–6337	Designed in this study
HEV-RPA-F2	TRCCGGCRGTGGTTTCTGGGGTGACMGGGT	5303–5332	Designed in this study
HEV-RPA-F3	CGGGTGGAATGAATAACATGTTCTTTTGCT	5146–5175	Designed in this study
HEV-RPA-F301	TCGGGTGGAATGAATAACATGTTCTTTTGC	5145–5174	Designed in this study
HEV-RPA-F302	TCGGGTGGAATGAATAACATGTTCTTTTGCT	5145–5175	Designed in this study
HEV-RPA-F303	ATCGGGTGGAATGAATAACATGTTCTTTTG	5144–5173	Designed in this study
HEV-RPA-F304	ATCGGGTGGAATGAATAACATGTTCTTTTGC	5144–5174	Designed in this study
HEV-RPA-F305	ATCGGGTGGAATGAATAACATGTTCTTTTGCT	5144–5175	Designed in this study
HEV-RPA-R1-1	ATRGYTATACCCTTRTCCTGCTGRGCRTTCTC	6444–6475	Designed in this study
HEV-RPA-R1-2	biotin-TGCTCATGTTGGTTRTCATAATCCTGR TAAC	6511–6541	Designed in this study
HEV-RPA-R2	TGGGMYTGGTCRCGCCAAGCGGAGCCRAGK	5441–5470	Designed in this study
HEV-RPA-R3	ATGCGAAGGGGTTGGTTGGATGAATATAGGG	5355–5385	Designed in this study
HEV-RPA-R301	GATGCGAAGGGGTTGGTTGGATGAATATAGG	5356–5386	Designed in this study
HEV-RPA-R302	RGATGCGAAGGGGTTGGTTGGATGAATATAG	5357–5387	Designed in this study
HEV-RPA-R303	CRGATGCGAAGGGGTTGGTTGGATGAATATA	5358–5388	Designed in this study
HEV-RPA-R304	TCRGATGCGAAGGGGTTGGTTGGATGAATAT	5359–5389	Designed in this study
HEV-RPA-R3001	GATGCGAAGGGGTTGGTTGGATGAATAT	5359–5386	Designed in this study
HEV-RPA-R3002	GATGCGAAGGGTTGGTTGGATGAATATA	5358–5385	Designed in this study
HEV-RPA-R3003	GATGCGAAGGGGTTGGTTGGATGAATATAG	5357–5386	Designed in this study
HEV-RPA-R3004	GATGCGAAGGGGTTGGTTGGATGAATATAGGG	5356–5386	Designed in this study
HEV-RPA-P1	TGGTGGYCARCTGTTTTACTCCCGCCCCGTCG(FAM-dT)(THF)(BHQ1-dT)CAGCCAATGGCGAGCC	6368–6414	Designed in this study
HEV-RPA-P3	FAM-CGGCGGTGGTTTCTGGGGTGACCGGGTTGATT(THF)TCAGCCCTTCGCCCTC-C3-spacer	5306–5354	Designed in this study
HEV-F (SN)	ACHCTRTTTAAYCTTGCTGAYAC	6306–6328	Qiao et al., 2008
HEV-R (SN)	CCTTRTCCTGTGAGCRTTCT	6390–6409	Qiao et al., 2008
HEV-P (SN)	FAM-CCGGACAGAATTGATTTCGTCGGC-BHQ1	6344–6367	Qiao et al., 2008

K:G or T, R:A or G, M:A or C, and Y:C or T. The location of primers/probes refers to the position in the genome of Chinese HEV strain swCH25 (GenBank accession number AY594199).

### qRT- RPA assay

The qRT-RPA assay was conducted in a 50-µl volume using the ZC BioScience™ exo kit (ZC BioScience, Hangzhou, China). The reaction mixture contained 25 μl of rehydration buffer, 2.5 μl of magnesium acetate (280 mM), 2.1 μl of each primer (10 μmol/L), 0.6 μl of exo probe (10 μmol/L), 5 μl of extracted nucleic acid, and 12.7 μl of ddH_2_O. The reaction tubes were immediately mixed and spun down. The qRT-RPA reactions were performed at 41°C for 20 min using a Genie III scanner device (OptiGene Limited, West Sussex, UK). The fluorescence signal was measured at an interval of every 20 s. Samples that yeilded an exponential amplification curve above the negative control threshold were considered positive.

### LFB RT-RPA assay

The LFB RT-RPA assay was performed in a 50-µl volume using the GenDxTM RT-LFB kit (GenDx, Suzhou, China). The reaction mixture contained 20 μl of rehydration buffer, 2.0 μl of magnesium acetate (280 mM), 2.1 μl of each primer (10 μmol/L), 0.6 μl of nfo probe (10 μmol/L), 5 μl of extracted nucleic acid, and 18.2 μl of ddH_2_O. To determine the optimal amplification temperature, the RPA reactions were performed on a metal bath incubator set at 39–45°C for 20 min. Thereafter, the reactions were performed at the optimal temperature for 5, 10, 20, and 30 min to determine the optimal incubation time. After the reaction, 5 μl of RPA products was mixed with 200 μl of ddH_2_O followed by lateral flow biosensor analysis (GenDx, Suzhou, China). The results were considered positive when both the test line and the control line were visible, negative when only the control line was visible, and invalid when the control line was invisible.

### Analytical specificity analysis

The specificity of the developed HEV-specific qRT-RPA and LFB RT-RPA assays was examined with 3.4 × 10^4^ copies of HEV standard RNA and 6.08 × 10^7^–5.15 × 10^9^copies of DNA or RNA of other important swine viruses including PRV, PCV2, PRRSV, CSFV, PPV, and FMDV. During the analysis, ddH_2_O was chosen to act as the negative control. All the samples were tested in triplicates.

### Analytical sensitivity analysis

The sensitivity analysis of the RT-RPA and RT-qPCR assays was performed using 10-fold serial dilutions of standard HEV RNA ranging between 3.4 × 10^6^ and 3.4 × 10^0^ copies/μl. Each run was repeated eight times. Meanwhile, 3.4 × 10^2^copies/μl of HEV RNA was serially diluted in twofold (one in one to one in four) and tested for the qRT-RPA and RT-qPCR assays in eight replicates, whereas 3.4 × 10^1^copies/μl of HEV RNA was serially diluted in twofold (one in one to one in four) and tested for LFB RT-RPA in eight replicates.

A Probit (predicted proportion positive) analysis (SPSS v22.0, Armonk, USA) with data of the positive samples from each of the eight replications was performed to establish the 95% limit of detection (LOD).

### Validation with raw pork livers

A total of 80 raw pork livers from different sources, including 14 HEV-positive livers and 66 randomly selected HEV-negative livers in qRT-PCR, were detected using the qRT-RPA and LFB RT-RPA assays, respectively.

## Results

### Screening of the optimal primer–probe combinations

Three rounds of primer screening were conducted in this study. Three forward and four reverse primers were designed and screened by using ZC BioScience™ basic kit in the primary screening of primer candidates. Among these, two primer pairs amplified HEV RNA (HEV-RPA-F1/R1-2 and HEV-RPA-F3/R3). The HEV-RPA-F3/R3 primer pair produced the greatest amount of amplification product and was selected for subsequent experiments (**Supplementary Figure S1A**). In the secondary screening of primer candidates, five forward (HEV-RPA-F301-F305) and four reverse primer candidates (HEV-RPA-R301-R304) were designed surrounding HEV-RPA-F3 and HEV-RPA-R3, respectively. Reverse primer HEV-RPA-R3 was selected to screen all six forward primers, and the primer with the best fluorescence signal was considered the best forward primer (HEV-RPA-F302) (**Supplementary Figure S1B**). Subsequently, HEV-RPA-F302 was selected to screen all the five reverse primers; HEV-RPA-R301 generated the best result (**Supplementary Figure S1C**). In the tertiary candidate screening, four reverse primer candidates (HEV-RPA-R3001-R3004) were designed surrounding HEV-RPA-R301 and were screend using the best forward primer HEV-RPA-F302. HEV-RPA-R301 was again used to generate the best amplification result (**Supplementary Figure S1D**). Therefore, HEV-RPA-F302/R301/P3 was subsequently used in the qRT-RPA assay.

In most cases, the similar primer and probe sequences with different residue modifications functioned effectively in both qRPA and LFB RPA assays. However, our data confirmed that HEV-RPA-F302/R301/P3 produced false-positive signals in the LFB RT-RPA assay. Therefore, the primer pair and probe HEV-RPA-F1/R1-2/P1 were selected for the HEV LFB RT-RPA assay.

### Performance of the RT-qRPA assay

**Figure 1:**
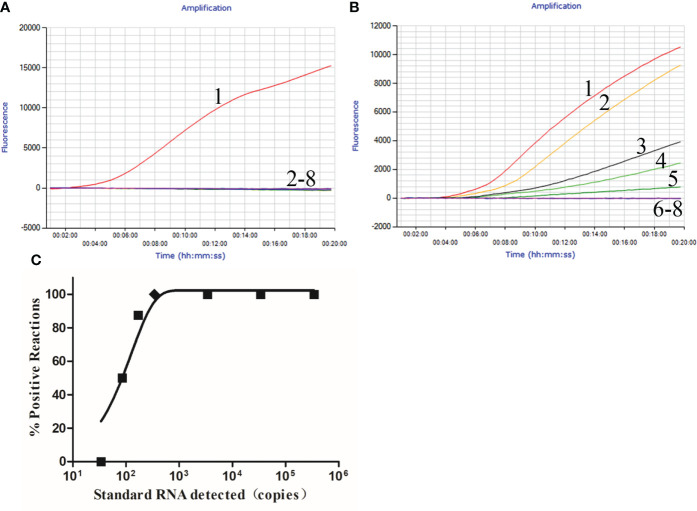
Performance of the HEV qRT-RPA assay. **(A)** Evaluation of the analytical specificity of the qRT-RPA assay. Lines 1–8: HEV, PRRSV, CSFV, FMDV, PRV, PCV2, PPV, and ddH_2_O. **(B)** Fluorescence amplification curves of qRT-RPA. Lines 1–7: 3.4 × 10^6^–3.4 × 10^0^copies/μl; line 8: ddH_2_O. **(C)** Probit regression analysis of the RT-qRPA assay using the data of the positive samples from each of the eight replicates. The limit of detection at 95% probability (154 copies/μl) is depicted by a rhomboid.

The specificity of RT-qRPA was evaluated using HEV and six other important swine-associated viruses. Consequently, only HEV RNA revealed a typical fluorescent signal in the RT-qRPA assay, whereas no fluorescent signals were obtained for the other six viruses (**Figure 1A**). Similar results were obtained in three repeats.

In the analytical sensitivity analysis, the HEV standard RNA dilutions ranging from 3.4 × 10^6^ to 3.4 × 10^0^ copies/μl were tested for eight replicates. For the RNA standards over 3.4 × 10^2^ copies/μl, both qRT-PCR (**Supplementary Figure S2A**) and qRT-RPA (**Figure 1B**) assays detected all eight replicates as positive. At 1.7 × 10^2^ copies/μl concentration, the qRT-RPA assay detected eight of eight *versus* seven of eight for the qRT-PCR assay; at 8.5 × 10^1^ copies/µl concentration, the qRT-RPA assay detected four of eight *versus* four of eight for the qRT-PCR assay. At 3.4 × 10^1^ copies/µl concentration, both qRT-PCR and qRT-RPA assays yielded negative outcomes in all eight replicates. The probit regression analysis revealed that the LOD of the qRT-RPA and qRT-PCR assays was 154 copies/µl (95%CI: 126 to 333 copies/µl) (**Figure 1C**) and 181 copies/μl (126–333 copies/µl), respectively (**Supplementary Figure S2B**).

### Optimization of LFB RT-RPA reaction conditions

**Figure 2:**
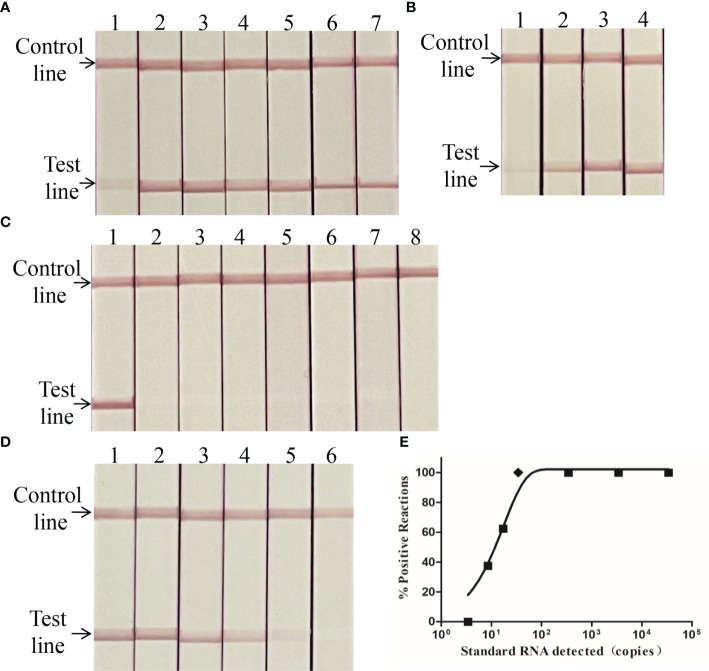
Performance of the HEV LFB RT-RPA assay. **(A)** Optimization of the lateral flow biosensor (LFB) RT-RPA reaction temperature. Lanes 1–7: 39, 40, 41, 42, 43, 44, and 45°C. **(B)** Optimization of the LFB RT-RPA incubation time. Lanes 1–4: 5, 10, 20, and 30 min. **(C)** Evaluation of the analytical specificity of the LFB RT-RPA assay. Lanes 1–8: HEV, PRRSV, CSFV, FMDV, PRV, PCV2, PPV, and ddH_2_O. **(D)** Evaluation of the analytical specificity of the LFB RT-RPA assay. Lanes 1–6: 3.4 × 10^5^–3.4 × 10^0^copies/μl. **(E)** Probit regression analysis of the LFB RT-RPA assay using the data of the positive samples from each of the eight replicates. The limit of detection at 95% probability (24 copies/μl) is depicted by a rhomboid.

HEV-specific LFBRT-RPA reaction conditions were optimized using 3.4 × 10^4^ copies/μl of HEV RNA as a template. As shown in **Figure 2A**, the LFB RT-RPA assay worked effectively at a temperature ranging between 39 and 45°C, and the brightest test lane was observed at 41°C. As shown in **Figure 2B**, the test line was extremely weak in reactions incubated for 5 min, and the test lines were clearer when the incubation duration was over 10 min. The assay performance was improved with a longer reaction time; there was no discernable difference after 20 min. Thus, 41°C and 20 min were set as the optimal conditions for the LFB RT-RPA assay.

### Performance of the LFB RT-RPA assay

For the specificity analysis of LFB RT-RPA, only HEV RNA was detected as positive, and no cross-reaction was noted with the other six swine-associated viruses (**Figure 2C**). The 10-fold serial dilutions of the HEV RNA that ranged between 3.4 × 10^5^ and 3.4 × 10^0^ copies/µl were used to evaluate the limit of detection. As shown in **Figure 2D**, the test line was observed between 3.4 × 10^5^ and 3.4 × 10^1^ copies/µl of HEV RNA, whereas five of eight, five of eight, and zero of eight were positive at concentrations of 1.7 × 10^1^, 8.5 × 10^0^, and 3.4 × 10^0^ copies/µl, respectively. According to Probit regression analysis, the LOD for the LFB RT-RPA assay was 24 copies/μl (95%CI: 20–57 copies/µl) (**Figure 2E**).

### Performance of RT-RPA assays on raw pork livers

For the 14 HEV RNA-positive livers in the qRT-PCR assay, the qRT-RPA and LFB RT-RPA detected 11 and 12 positive samples, respectively. All 66 randomly selected negative samples were HEV RNA-negative in both qRT-RPA and LFB RT-RPA assays (**Table 2**). The coincidence rate between the test results of RT-RPA and RT-qPCR was 96.3%, whereas the coincidence rate between the test results of LFB RT-RPA and RT-qPCR was 97.5%. No significant difference was noted between the qRT-RPA and qRT-PCR assays (*p* = 0.25). No significant difference was also found between LFB RT-RPA and qRT-PCR assays (*p* = 0.50). Furthermore, the qRT-RPA and RT-qPCR assays were significantly in agreement (kappa = 0.858 at 95%CI). The LFB RT-RPA and qRT-PCR assays were also significantly in agreement (kappa = 0.908 at 95%CI). The above-mentioned data show that the developed HEV-specific RT-RPA assays had a similar diagnostic performance with qRT-PCR on clinical samples (**Table 3**).

**Table 2:** Detection results of the HEV RNA-positive samples.

Number	Sources	qPCR (cycle threshold, Ct)	qRT-RPA (time threshold, Tt, mm:ss)	LFB RT-RPA	Genotype
1	BsDC, Linzhang, Handan	30.23	07:26	+	4d
2	BsDC, Wuan, Handan	30.44	07:37	+	4d
3	BsDC, Wuan, Handan	32.17	07:56	+	4d
4	BsDC, Wuan, Handan	34.28	06:49	+	4d
5	BsDC, Wuan, Handan	32.55	09:01	+	4d
6	BsDC, Wuan, Handan	32.55	07:35	+	4d
7	BsDC, Wuan, Handan	37.04	–	+	4d
8	BsDC, Wuan, Handan	32.19	09:30	+	4d
9	BsDC, Baoding	37.46	–	–	4d
10	BsDC, Baoding	34.22	–	+	4d
11	BsDC, Shijiazhuang	34.58	08:01	–	4d
12	BsDC, Shijiazhuang	32.23	09:56	+	4d
13	Slaughterhouse, Chengde	33.16	10:14	+	4d
14	Slaughterhouse, Chengde	32.87	09:48	+	4d

BsDC, Bio-safety Disposal Centers for Dead Livestock and Poultry; +, positive; -, negative.

**Table 3:** Comparative performances of the qRT-PCR, qRT-RPA, and LFB RT-RPA assays for the detection of HEV RNA in raw pork livers.

Assay		qRT-PCR			Kappa	*p*-value	CR
	Positive	Negative	Total			
qRT-RPA	Positive	11	0	11	0.858	0.25	96.3%
	Negative	3	66	69
	Total	14	66	80
LFB RT-RPA	Positive	12	0	12	0.908	0.50	97.5%
	Negative	2	66	68
	Total	14	6	20

CR, coincidence rate.

## Discussion

Since its first description in 2006, RPA technology has received significant research attention. At present, RPA is widely used in the detection of pathogens, including bacteria, viruses, and parasites. It fosters innovation in the field of isothermal nucleic acid amplification technology (Euler et al., 2013; Dobnik et al., 2016; Liu et al., 2019).

Suitable primers and probes are critical for the success of an RPA assay. So far, software for designing RPA primers is unavailabe. The RPA primer screening process is similar to PCR, *i*.*e*., it involves the selection of the target region, designing the primer candidates, and screening for the best candidate (Lobato and O'Sullivan, 2018). Hairpin structure, primer dimers, and primer–primer interactions should be avoided (Li et al., 2021). Nevertheless, we detected several mutations in the *ORF2* gene sequence of HEV. Moreover, the continuous conserved region was short and scattered, limiting the selection of primers and probes for the RPA assay. Thus, the primer conservation was the only factor in the initial design of primers, and the amplification efficiency of primers was experimentally confirmed. After three rounds of screening, HEV-RPA F302/R301/P3 was selected for the qRT-RPA assay, whereas HEV-RPA F1/R1-2/P1 was selected for the LFB RT-RPA assay.

**Figure 3:**
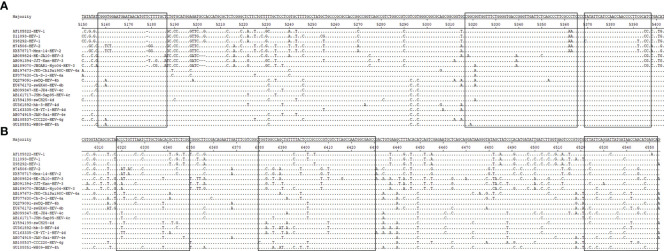
Primer and probe positions within *ORF2* gene of HEV genotypes 1–4. The unfilled boxes represent the primer regions used in this study. Nucleotide residues that match the majority are indicated by dots; nucleotide deletions are indicated by dashes. **(A)** Primers and probes used for the RT-qRPA assay. **(B)** Primers and probes used for the LFB RT-RPA assay.

Furthermore, the designed primers and probes were compared using the DNASTAR software with representative HEV genotypes 1–4. In the qRT-RPA assay, four to five nucleotide mismatches were detected in HEV-1, 10 nucleotide mismatches were detected in HEV-2, six to seven nucleotide mismatches were detected in HEV-3, and zero to three nucleotide mismatches were detected in HEV-4. In the LFB RT-RPA assay, five to nine nucleotide mismatches were detected in HEV-1, 12 to 13 nucleotide mismatches were detected in HEV-2, six to nine nucleotide mismatches were detected in HEV-3, and four to nine nucleotide mismatches were detected in HEV-4. Previous studies have shown that RPA can tolerate five to nine mismatches in primers and probes without affecting its performance (Boyle et al., 2013; Liu et al., 2019). In this regard, it was difficult for the two RPA assays that we developed to detect HEV-2, but in theory, these would perform well in detecting the other three genotypes (**Figure 3**). All isolated HEV strains in this study belonged to the genotype 4d, with four to nine nucleotide mismatches from the primers and probes of the RT-RPA assays established in this study (unpublished data). This indicates that the primers and probes of the RPA method are highly resistant to mutations. Nevertheless, further validation studies are necessary for the other HEV genotypes.

After optimizing the reaction conditions, the qRT-RPA and LFB RT-RPA assays display a similar performance to the qRT-PCR assay (Qiao et al., 2008), whereas the LOD obtained from qRT-RPA is distinct with the LFB RT-RPA assay in this study. There are two possible reasons for this phenomenon. One reason is the use of different primer and probe sequences and different nucleases (the qRT-RPA assay uses exonuclease, while the LFB RT-RPA assay uses endonuclease), and the other reason is the different detection methods for the amplification products. The performance of the RT-RPA assays on the raw pork livers of different sources was slightly lower than that of the qRT-PCR assay. However, the RT-RPA demonstrated distinct advantages of rapidness and convenience, suggesting that the developed RT-RPA assays can be used as alternative detection techniques for HEV. Three samples were negative in the RT-RPA assays but weakly positive in qRT-PCR, with Ct values of approximately 34.22–37.04, which contained low amounts of HEV RNA. Our findings are similar to previous reports that detected *Mycoplasma hyopneumoniae*, bovine ephemeral fever virus, and peste des petits Ruminant’s virus through RPA assays (Hou et al., 2018; Yang et al., 2018; Davi et al., 2019). Therefore, target gene concentration should be increased by increasing the sample content in the reaction system to improve the detection performance of the clinical samples.

RPA technology has many incomparable strengths over qPCR technology. Firstly, RPA operates at a relatively low temperature, *i*.*e*., body temperature, water bath, or heating blocks (Crannell et al., 2014; Cherkaoui et al., 2021). Secondly, RPA can tolerate up to nine mismatches, hence increasing the selection of primers for viruses with robust nucleic acid sequence mutations (Boyle et al., 2013). Thirdly, RPA reagents are freeze-dried, and the RPA kit can be stored at room temperature for up to 6 months (Chandu et al., 2016). The above-mentioned benefits enable the RPA assays to be more suitably used at point-of-care (POC) or underequipped laboratory diagnosis and in resource-limited settings or field diagnosis of various pathogens. However, it is necessary to open the reaction tube before lateral flow biosensor analysis. This process may carry over aerosol contamination in fields. In order to reduce potential contamination, the laboratory should have a rigorous partition, and UV irradiation or DNase treatment should be used frequently. In addition, the reaction tubes should be carefully opened and closed, and gloves should be frequently changed during RPA operation.

In conclusion, the RPA assays developed in this work have significant potential for POC detection of HEV and can therefore be used in the field.

## Data availability statement

The original contributions presented in the study are included in the article/**Supplementary Material**. Further inquiries can be directed to the corresponding authors.

## Ethics statement

The animal study was reviewed and approved by the Medical Ethics Committee of Hebei Medical University.

## Author contributions

JinW and XX conceptualized and designed the experiments. KW collected the samples and performed the experiments. JiaW, CZ, XS, and LL collected the samples and analyze the data. JinW and KW wrote and revised the manuscript. All authors contributed to the article and approved the submitted version.

## Funding

This work was supported by the Science and Technology Program of Hebei province (19977719D, 19226612D, and 20277723D).

## Conflict of interest

The authors declare that the research was conducted in the absence of any commercial or financial relationships that could be construed as a potential conflict of interest.

## Publisher’s note

All claims expressed in this article are solely those of the authors and do not necessarily represent those of their affiliated organizations, or those of the publisher, the editors and the reviewers. Any product that may be evaluated in this article, or claim that may be made by its manufacturer, is not guaranteed or endorsed by the publisher.
