# Supplementary material for: Rapid and direct detection of hepatitis E virus in raw pork livers by recombinase polymerase amplification assays
Source: Front Cell Infect Microbiol. 2022 Sep 5;12:958990. doi: 10.3389/fcimb.2022.958990 (PMC9483107; doi:10.3389/fcimb.2022.958990)
Supplement: Supplementary file 1 [file DataSheet_1.docx]

***Supplementary Material***

**
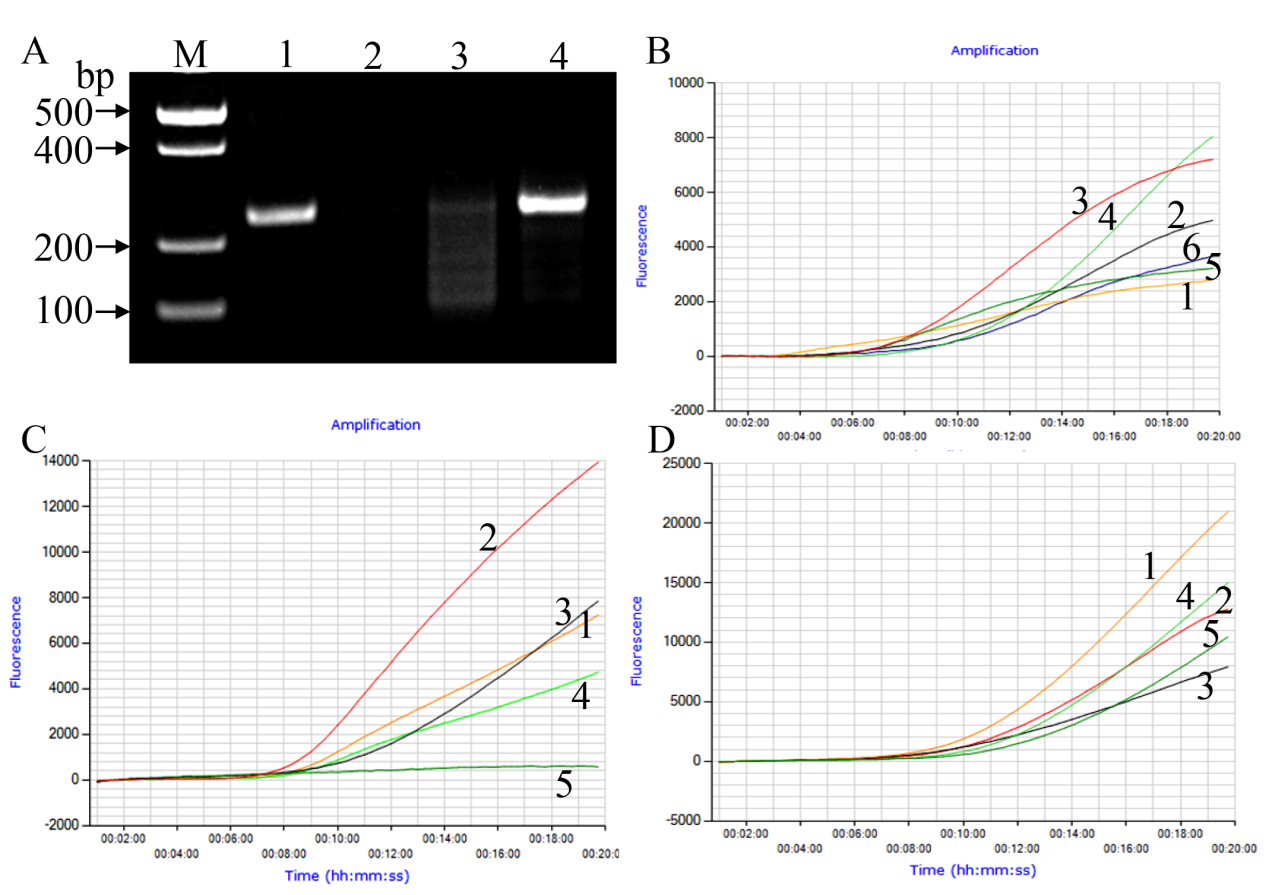
**

**Supplementary Figure 1.** Screening of the primers. (A) Primary candidate screen. Lane M: DNA Marker. Lanes 1-4: HEV-RPA-F1/R1-2, HEV-RPA-F1/R1-1, HEV-RPA-F2/R2, HEV-RPA-F3/R3. (B) The secondary candidate screen. Six forward primers were selected by reverse primer R3. Lanes 1-6: HEV-RPA-F3/R3, HEV-RPA-F301/R3, HEV-RPA-F302/R3, HEV-RPA-F303/R3, HEV-RPA-F304/R3, HEV-RPA-F305/R3. (C) The secondary candidate screen. Five reverse primers were selected by forward primer HEV-RPA-F302. Lanes 1-5: HEV-RPA-F302/R3, HEV-RPA-F302/R301, HEV-RPA-F302/R302, HEV-RPA-F302/R303, HEV-RPA-F302/R304. (D) The tertiary candidate screen. Five reverse primers were selected by forward primer HEV-RPA-F302. Lanes 1-5: HEV-RPA-F302/R301, HEV-RPA-F302/R3001, HEV-RPA-F302/R3002, HEV-RPA-F302/R3003, HEV-RPA-F302/R3004.


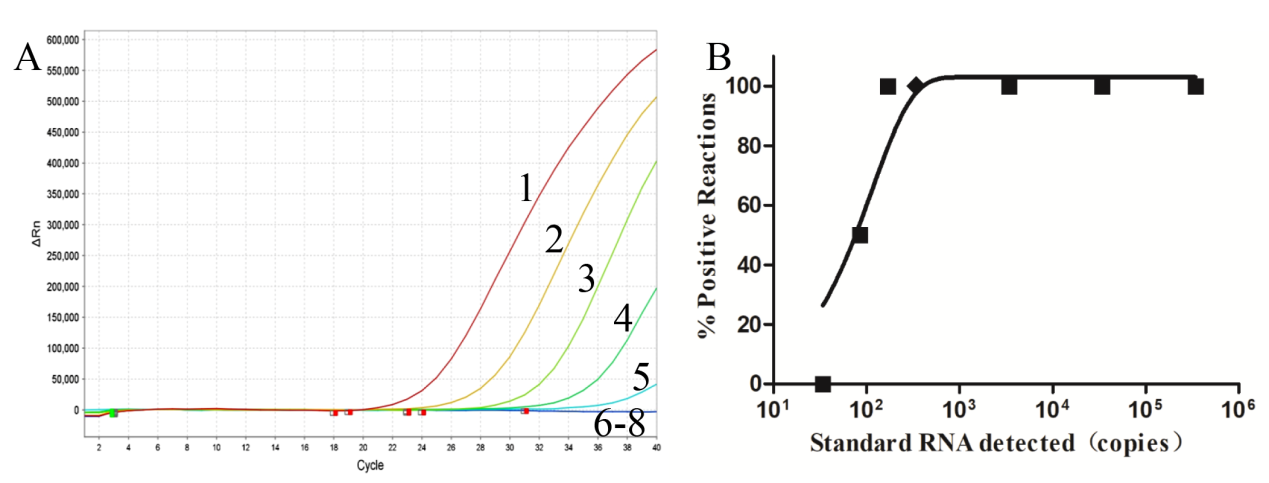


**Supplementary Figure 2.** The analytical sensitivity of HEV RT-qPCR. (A) The fluorescence amplification curves of qRT-PCR. Lines 1–7: 3.4 × 10^6^–3.4 × 10^0^ copies/µL; Line 8: ddH_2_O. (B) Probit regression analysis of the RT-qPCR assay using the data of the positive samples from each of the 8 replicates. The limit of detection at 95% probability (181copies/μL) is depicted by a rhomboid.
